# Transition versus Continuous Slope Walking: Adaptation to Change Center of Mass Velocity in Young Men

**DOI:** 10.1155/2018/2028638

**Published:** 2018-06-19

**Authors:** Yoon No Gregory Hong, Jinkyu Lee, Choongsoo S. Shin

**Affiliations:** ^1^Department of Mechanical Engineering, Colorado School of Mines, Golden, CO 80410, USA; ^2^Department of Mechanical Engineering, Sogang University, 35 Baekbeom-ro, Mapo-gu, Seoul 04107, Republic of Korea

## Abstract

During continuous uphill walking (UW) or downhill walking, human locomotion is modified to counteract the gravitational force, aiding or impeding the body's forward momentum, respectively. This study aimed at investigating the center of mass (COM) and center of pressure (COP) velocities and their relative distance during the transition from uphill to downhill walking (UDW) to determine whether locomotor adjustments differ between UDW and UW. Fourteen participants walked on a triangular slope and a continuous upslope of 15°. The kinematics and COPs were obtained using a force plate and a motion capture system. The vertical velocity of the COM in the propulsion phase, the horizontal distance between the COM and COP at initial contact, and the duration of the subphases significantly differed between UDW and UW (all *p* < 0.05). Compared with the results of UW, longer durations and the deeper downward moving COM in the propulsion phase were observed during UDW (all *p* < 0.05). Additionally, a shorter horizontal distance between the COM and COP at initial contact was associated with a slower vertical COM velocity in the propulsion phase during UDW. The reduced velocity is likely a gait alteration to decrease the forward momentum of the body during UDW.

## 1. Introduction

Activities of daily living occasionally require pedestrians to walk on slopes, which places more mechanical demand on lower-extremity joints compared with level walking [1]. During slope walking, the neuromuscular system must be controlled to maintain body stability and ensure the proper movement of the body's center of mass (COM) [2–4]. The control strategy is altered when facing different slope angles, with different joint kinematics and kinetics patterns [5, 6]. In particular, implementing motor control for slope walking is more challenging for children, the elderly, and patients with musculoskeletal disorders; thus, walking on a slope is highly associated with the risk of falls [7–9].

**Figure 1:**
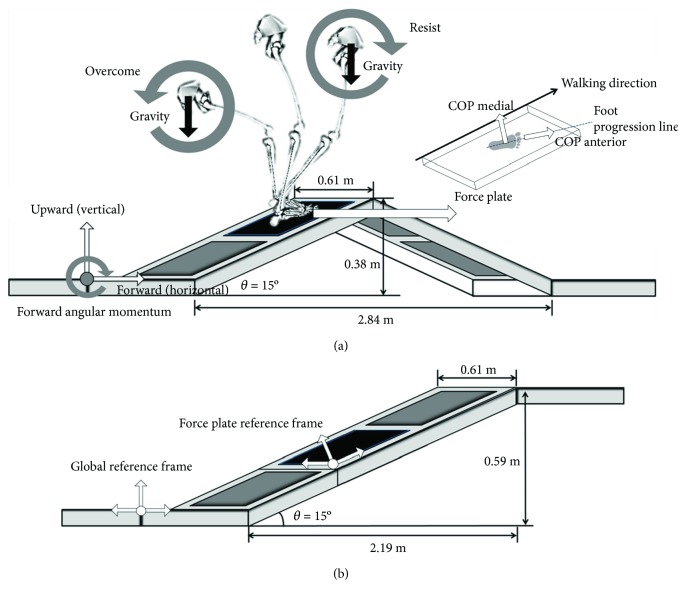
Custom-made slope walkway in a diagonal view. (a) Illustration of the lower extremity during walking at the up-down transition on a triangle-shaped slope. When the body's center of mass (COM) is behind the center of pressure (COP) at an early stance phase, the gravitational force generates a backward angular momentum. To prevent backward falls, the backward angular momentum must be resisted. When the body's COM is ahead of the COP at a late stance phase, the gravitational force generates a forward angular momentum. To prevent forward falls, the forward angular momentum must be resisted. The anterior-posterior axis of the COP is coincident with the longitudinal axis of the foot progression line. The medial-lateral axis of the COP is perpendicular to the anterior-posterior axis of the COP. (b) Continuous uphill walkway. To recalculate the measurements of the 3 axes of the ground reaction force, a global reference frame was established on the ground, and the force plate had its own local reference frame.

Many previous studies have reported the effects of the slope angle on human locomotion [10–13]. During slope walking, the body needs to actively counteract the gravitational effect, which pulls the body downward in the opposite vertical direction during uphill walking (UW) but pushes the body downward in the same vertical direction during downhill walking (DW). Because these gravitational effects could be associated with a backward fall and a forward fall during UW and DW, respectively, it is important to consider the gravitational effects on the forward momentum of the body to evaluate the locomotion during slope walking (Figure 1(a)). In particular, during the transition from uphill to downhill walking (UDW), if the forward momentum generated is equal to that generated during continuous UW, the body's forward momentum could cause a forward fall when confronting the downhill surface. Although the current foot step is still placed on the uphill slope, the locomotor adjustment might need to be modified to manage the balance between the current uphill and the upcoming downhill (Figure 1(a)).

A proper COM movement relative to the center of pressure (COP) is necessary to manage the gravitational effects on the body's forward momentum during UDW. Because the COM position and COM velocity relative to the COP are associated, there is a corresponding upper and lower boundary of the COM velocity at a certain COM position to maintain dynamic stability during walking [14, 15]. For a specific COM position, a COM velocity beyond this boundary initiates a fall [15]. Moreover, the movement of the COM relative to the COP strongly influences the direction and magnitude of the moment at the lower-extremity joints and the COM [16] and can provide insight into the postural challenges of various motions [17, 18]. The primary difference between the UW and UDW conditions is whether the next step is a continuous uphill step (Figure 1(b)) or a transition from uphill to downhill (Figure 1(a)) after stepping on the same slope, with a force plate embedded. Compared to the locomotion during UW, the locomotion of the last step on the uphill surface during UDW is expected to achieve a slower COM velocity because if the same COM velocity as UW is maintained during UDW, it could cause a forward fall during the transition. Additionally, minimizing the horizontal COM-COP distance is an effective strategy to achieve sufficient anterior-posterior stability [19]. This strategy can be used to prevent forward falling during UDW. Thus, one can expect that the locomotion of the last step on the uphill surface during UDW would have a slower COM velocity and a shorter horizontal COM-COP distance.

People often encounter transitions between differently sloped terrains. Given the need for evidence-based practice in the clinic, more information about motor control during the transition between differently sloped terrains is required, which may help clinicians to assess and treat patients walking not only on continuous slopes but also transitioning between differently sloped terrains. Thus, the current study aims at investigating the anterior-posterior and medial-lateral velocities of the COP relative to the foot progression line (Figure 1(a)), the horizontal and vertical velocities and the position of the COM (Figure 1(b)), the horizontal distance between the COM and COP (COM-COP distance) (Figure 1(b)), and the duration of the subphases (the loading response, the midstance, and the propulsion phases) during the stance phase of UDW compared with those of UW. It was hypothesized that (1) the horizontal and vertical COM velocities would be slower during the propulsion phase during UDW than those during UW and (2) the horizontal COM-COP distance would be modified during UDW compared with that during UW.

## 2. Materials and Methods

Fourteen participants were recruited (age: 22.8 (2.5) years, height: 173.8 (3.4) cm, mass: 66.5 (5.3) kg, BMI: 22.0 (1.9) kg/m^2^). All participants were healthy males without any pain or history of lower-extremity musculoskeletal injuries that require surgery. All participants signed an informed consent form approved by the institutional review board.

A triangular walkway (Figure 1(a)) was constructed to mimic an outdoor terrain combining uphill and downhill walking conditions. The walkway begins with an inclination angle of 15° and is followed by a declination angle of 15°. A force plate was securely embedded into the inclined walkway for the UDW (Figure 1(a)) and for continuous UW (Figure 1(b)). The subjects started 5 steps away from the slope to have a comfortable walking speed with own-step length and were asked to step on the force plate with their dominant limb [20, 21]. The dominant leg was defined as the more comfortable leg for kicking a ball [22]. Prior to the actual experimental trials, each participant was instructed to perform several practice trials for both walkway conditions at a comfortable walking speed [20]. A 3D motion capture system equipped with 8 infrared cameras (5 Eagle, 2 Hawk, and 1 Raptor, Motion Analysis Corp., Santa Rosa, CA, USA) was used to record the motion of the markers for the calculation of knee and ankle joints at a sampling rate of 200 Hz during walking on the triangular slope. Reflective markers (*Ф*12.5 mm spheres) were attached to the following anatomical bony landmarks in dominant limb: the bilateral anterior superior iliac spines, the sacrum, the greater trochanter, the midpoint of the femur, the lateral and medial epicondyles of the femur, the lateral and medial plateaus of the tibia, the midpoint of the tibia, the lateral and medial malleoli, the calcaneus, and the first and fifth metatarsal heads [20]. A force plate (9260AA6, Kistler, Winterthur, Switzerland) was embedded in the sloped walkway and used at a sampling rate of 1200 Hz, synchronized with the motion capture system [20].

**Figure 2:**
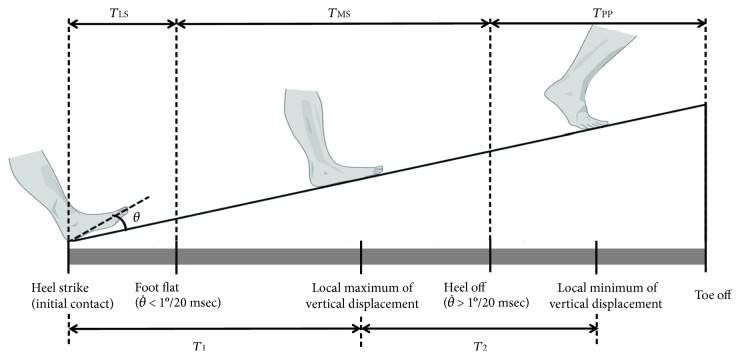
Stance subphases and gait events. The stance phase was divided into three subphases: the loading response (from initial contact to foot flat, *T*_LS_), the midstance (from foot flat to heel off, *T*_MS_), and the propulsion (from heel off to toe off, *T*_PP_) phases. *T*_1_: duration from initial contact to the local maximum of the vertical COM displacement. *T*_2_: duration from the local maximum of the vertical COM displacement to the local minimum of the vertical COM displacement.

The heel strike (initial contact) and toe-off time were identified using the force plate data. The stance phase was divided into three subphases: the loading response (from initial contact to foot flat), the midstance (from foot flat to heel off), and the propulsion (from heel off to toe off) phases (Figure 2). The initial contact during walking was identified by determining the first frame in which the vertical ground reaction force exceeded 20 N. The foot flat was identified by determining the first frame in which the foot segment rotated by less than 1° per 0.02 s in the sagittal plane of the global coordinate system. Finally, the heel off was identified by determining the first frame in which the foot segment rotated by more than 1° per 0.02 s after the foot flat [23].

The measured kinematic and kinetic data were filtered using a zero-lag, fourth-order Butterworth low-pass filter, with a cutoff frequency of 10 Hz. To calculate the joint kinematics, the coordinate systems for each body segment were defined using the modified Helen Hayes marker set [24]. The femoral and tibial coordinate systems for the knee joint were defined using a previously described method [25]. The ankle joint coordinate system was defined using a similar procedure to the knee joint. The superior-inferior (SI) axis was the cross product of the two vectors, which are from the heel to the first metatarsal head and from the heel to the fifth metatarsal head as a normal to the sole. The temporary medial-lateral (ML) axis was the cross product of the two vectors, which are from the heel to the midpoint of a line between the medial and lateral malleoli and from the heel to any point that lies on the SI axis. The SI axis and the temporary ML axis produced the anterior-posterior (AP) axis. Finally, to complete the orthogonal coordinate system, the cross product of the AP and SI axes was performed. The knee joint center was estimated to be at the midpoint of a line between the medial and lateral tibial plateau. The ankle joint center was calculated to be at the midpoint of markers placed on the medial and lateral malleoli. Knee and ankle joint angles were calculated using Euler angle rotations of the tibia relative to the femur and of the foot relative to the tibia [26]. The force data (ground reaction force) were measured in a force plate reference frame based on the slope angle (15°) and were transformed relative to the global reference frame to calculate inverse dynamics.

The knee and ankle kinetic values were obtained by combining the kinematic and ground reaction force data with anthropometric data and solving the Newton-Euler equations using inverse dynamics [27]. Joint power was defined as the product of joint angular velocity and moment. Joint work was taken as the integral of joint power over time during the stance phase. The power and work were normalized using the subject's body weight and height (W/(BW^∗^Ht)) and (J/(BW^∗^Ht)) [2]. The peak power of the knee and ankle was calculated during the stance phase.

**Figure 3:**
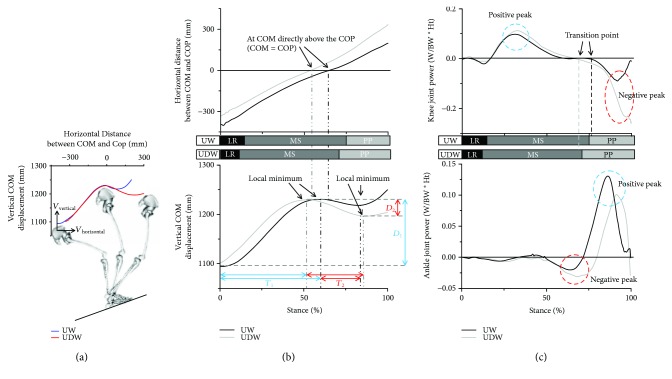
Illustration of the COM and COP movements and Ensemble curves for the joint power. (a) Schematic figure of lower-extremity walking on a slope with the mean ensemble curve for the vertical COM displacement versus COM-COP distance during stance. (b) Mean ensemble curves for the vertical COM displacement and the COM-COP distance during stance. *T*_1_: duration from the initial contact to the local maximum of the vertical COM displacement. *T*_2_: duration from the local maximum of the vertical COM displacement to the local minimum of the vertical COM displacement. *D*_1_: displacement of the vertical COM from the initial contact to the local maximum. *D*_2_: displacement of the vertical COM from the local maximum to the local minimum. (c) Mean ensemble curves for the ankle and knee joint power. The majority of the negative knee joint power and the positive ankle joint power were observed during the propulsion phase.

The horizontal COM-COP distance was the vector distance from the COP to the COM in the horizontal plane and was described by the COM position relative to the COP in a global reference frame, with a value of zero indicating a COM position directly above the COP and a positive value indicating a COM position anterior to the COP (Figures 3(a) and 3(b)). The vertical position of the COM was described relative to the global reference frame. Only the anterior/posterior and medial/lateral velocity of the COP was described relative to the foot progression line, which was a vector from the minimum point to the maximum point of the COP in the reference frame on the force plate (15° angle from the ground, Figure 1(a)). The average velocities of the COM and COP during each subphase were acquired by calculating the first derivatives of the offset displacement data within each subphase. The sagittal plane movement of the greater trochanter of the femur was used as an approximation of the COM movement [26, 28, 29]. The trajectory of the greater trochanter from initial contact to toe off in the horizontal and vertical planes of the global coordinate system was obtained from the location of the greater trochanter. The position of the COP was obtained from the force plate data. All time values, except the stance time, were expressed as a percentage of the duration of the stance phase.

For all parameters, paired one-tailed *t*-tests were performed to determine significant differences between UDW and UW at a significance level of 0.05. In addition, regression analysis was performed to investigate the relationship between the COM-COP distance at initial contact and the vertical velocity of the COM. All dependent variables were evaluated for normality using the Shapiro-Wilk *W* test, and the results did not indicate any violation of the normality assumption. Statistical analyses were performed using MATLAB version R2011a (MathWorks, Natick, MA, USA).

## 3. Results

Compared with COM velocities during UW, COM moved faster in both the horizontal (*p* = 0.012, Table 1) and vertical directions (*p* < 0.01, Table 1) during the loading response phase, and moved slower in the vertical direction through midstance (*p* = 0.019, Table 1) to propulsion phase (*p* < 0.01, Table 1) during UDW. During UDW, the COM was closer to the COP in the horizontal direction at initial contact (*p* = 0.024, Table 1, Figures 3(a) and 3(b)), and this difference of COM-COP distances between UW and UDW at initial contact was maintained through the stance phase. Thus, the COM was farther from the COP at toe off (*p* < 0.01, Table 1, Figures 3(a) and 3(b)). During UDW, the COM was closer to the COP in the horizontal direction at initial contact (*p* = 0.024, Table 1, Figures 3(a) and 3(b)), and this difference of COM-COP distances between UW and UDW at initial contact was maintained through the stance phase. Thus, the COM was farther from the COP at toe off (*p* < 0.01, Table 1, Figures 3(a) and 3(b)).

Even though the stance time was not significantly different between UDW and UW (*p* = 0.183, Table 1), the duration of the subphases was significantly different between UDW and UW. In the duration of the subphases, the COM reached the local maximum earlier (*T*_1_, *p* < 0.01, Table 1, Figure 3(b)) during UDW. Then the COM moved downward deeper (*D*_2_, *p* < 0.01, Table 1 and Figure 3(b)) with a longer duration (*T*_2_, (*p* < 0.01, Table 1 and Figure 3(b)) during UDW. Also, the duration of propulsion was significantly longer during UDW (*p* = 0.044, Table 1). In ankle and knee joint peak power and work, both conditions showed that peak positive ankle power occurred at the propulsion phase with a significantly decreased value during UDW (*p* < 0.01, Table 2 and Figure 3(c)). The peak positive knee power occurred first with no significant differences, and then the peak negative power appeared with a significantly increased value during UDW (*p* < 0.01, Table 2 and Figure 3(c)). The positive ankle work and the negative knee work were significantly decreased and increased during UDW, respectively.

**Figure 4:**
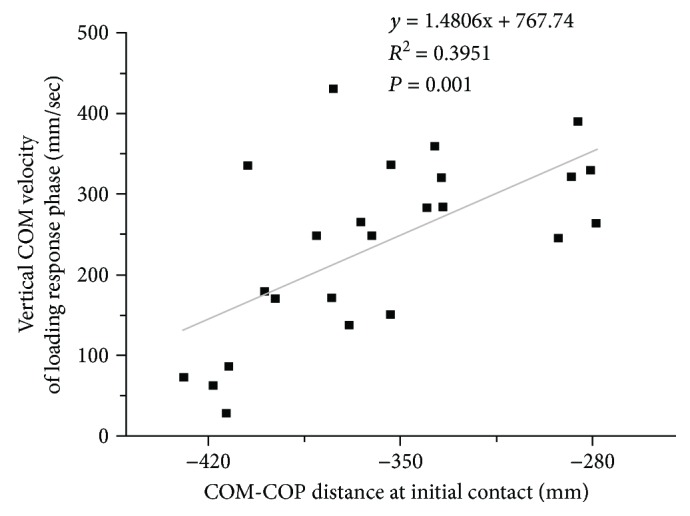
Relation between vertical COM velocity and COM-COP distance. Scatter plot for the vertical COM velocity of the loading response phase versus the COM-COP distance at the initial contact. The regression analysis showed that the COM-COP distance at the initial contact was significantly positively correlated with the vertical velocity of the COM. Linear regression lines and *R*^2^ values are provided for the data of 14 subjects.

Regression analysis showed that the COM-COP distance at the initial contact was significantly positively correlated with the vertical velocity of the COM (*R*^2^ = 0.395, *p* < 0.01, Figure 4). As the participants showed a shorter COM-COP distance at initial contact, the greater vertical COM velocity was revealed.

## 4. Discussion

This study aimed at determining whether the locomotor adjustment in relation to the movement of the COM and COP differs during UDW and UW. As predicted, a significantly slower vertical COM velocity during the propulsion phase was observed during UDW. The locomotor adjustments in this study, which are the significantly shorter horizontal COM-COP distance at initial contact and the significantly shorter time to reach the peak vertical position of the COM, appear to help the participants achieve a slower vertical COM velocity during the propulsion phase during UDW. Therefore, these results suggest that different locomotor adjustments are needed during UDW than during UW to reduce the vertical COM velocity during the propulsion phase.

In agreement with hypothesis 1, a significant decrease in the vertical COM velocity during the propulsion phase was observed during UDW compared with that during UW. This result indicates that the participants reduced their forward momentum during the propulsion phase of UDW. During DW, the forward momentum might be increased due to gravity. Because this increased forward momentum impairs the control of anterior-posterior and medial-lateral movements and increases the risk of falling during DW [30], one should reduce the body's forward momentum during the propulsion phase while preparing to transition during UDW. The momentum is the product of the body's mass and velocity. The change in COM velocity during walking results from the interaction between the internal muscle forces and the external gravitational force. The mechanical power and work generated by the muscles in the lower-extremity joints are the fundamental source that changes the body's COM. The gravitational force contributes to the forward acceleration of the COM during the propulsion phase in UDW. Nevertheless, our results showed that the vertical COM velocity decreased during the propulsion phase in UDW, which implies that more mechanical power and work (produced by muscular effort) were utilized to resist the gravitational force during UDW. Similarly, our joint power and work results showed that muscular effort was expended to resist the gravitational force during UDW. A significantly decreased peak positive ankle power and positive ankle work and a significantly increased peak negative knee power and negative knee work were observed during UDW compared with those observed during UW. More negative joint work and less positive joint work seemed to resist more (or assist less) the body's forward progression during the propulsion phase. Therefore, the increased negative knee power and work and decreased positive ankle power and work are needed to decrease the vertical COM velocity during UDW compared with UW to decrease the body's forward momentum.

For the power curve pattern, our results showed that the peak knee negative power and the peak positive ankle power occurred at the terminal stance, which were similar to the results of a previous study on 10° UW [31]. Additionally, our results showed that peak positive knee power was approximately 31.0% that of the stance phase, which agrees with previous results (29.0%, McIntosh et al. [31]). Moreover, the peak joint power showed comparable values with those from a previous study [31]. In our study, the peak knee negative power at the terminal stance significantly differed between UW and UDW. This difference could be related to a different vertical COM movement at the terminal stance between UW and UDW. As the vertical COM during UDW reaches the same peak height as it does during UW, it lowers more at the terminal stance of UDW than it does during UW and maintains this position until toe off; therefore, the participants might need more knee negative power to keep lowering the body and to maintain balance at the terminal stance during UDW.

Although there was no significant difference in the stance time between UDW and UW, the longer duration of *T*_PP_ and *T*_2_ helps participants decrease the vertical COM velocity during the propulsion phase during UDW. Because work is the time integral of the power, the duration of the power produced by the muscles is important. Most of the negative knee joint power was observed during *T*_PP_ (Figure 3(c)). Thus, a longer duration of *T*_PP_ with a greater negative peak knee joint power likely generates greater negative work during UDW. Furthermore, the starting point of *T*_PP_ appears to be a transition point from a positive to negative knee joint power (Figure 3(c)). The earlier generation of the negative knee joint power together with the earlier starting point of *T*_PP_ during UDW appears to suppress and delay the generation of a positive ankle joint power. Within *T*_2_, the COM exhibited a downward movement during both tasks (Figure 3(b)). In particular, a greater vertical displacement of the COM must occur during UDW than during UW because of the next downhill step. If the displacement is fixed, a longer duration of the time of change would decrease the velocity. Thus, the significantly longer duration of *T*_2_, together with the greater necessary amount of vertical displacement of the COM (*D*_2_), decrease the vertical velocity of the COM during UDW (Figure 3(b)). These results suggest that longer *T*_PP_ and *T*_2_ phase durations are needed to decrease the vertical velocity of the COM to minimize the body's forward momentum and to prevent a forward fall.

These longer *T*_PP_ and *T*_2_ durations during UDW appear to be achieved through the significantly shorter horizontal COM-COP distance of the initial contact during UDW than that during UW. Two factors may support this result. First, the first peak vertical position of the COM occurs approximately when the COM passes directly above the COP during walking (Figures 3(a) and 3(b)). Thus, a shorter horizontal COM-COP distance at initial contact could cause the first vertical peak position to be reached earlier, which indicates a shorter duration of *T*_1_. Second, the regression analysis showed that the position of the COM (relative to the COP) at initial contact was significantly positively correlated to the vertical velocity of the COM, which indicates that the change in the COM velocity is accompanied by a modification of the horizontal COM-COP distance. This finding indicates that the reduced horizontal COM-COP distance at initial contact allows the participants to achieve a greater vertical velocity of the COM during the loading response phase during UDW. Before *T*_PP_, this vertical COM velocity in the loading response is the only COM velocity that is significantly greater during UDW than that during UW. Both explanations suggest that the shorter horizontal COM-COP distance at initial contact helps the participants have a shorter duration *T*_1_ and longer duration *T*_PP_ and *T*_2_ during UDW.

The significantly shorter horizontal COM-COP distance at initial contact during UDW could result from a proactive strategy to not only achieve longer *T*_PP_ and *T*_2_ durations but also increase the attentiveness and the anterior-posterior body stability during UDW. When encountering a transition or perturbation, one is expected to be more attentive to ensure body stability. Minimizing the horizontal COM-COP distance is an effective strategy to achieve sufficient anterior-posterior stability with minimum control effort [19]. Thus, the significantly shorter horizontal COM-COP distance at initial contact is likely an adaptation to maintain body stability during UDW. This locomotor adjustment at initial contact must be achieved through the previous gait cycle. A previous study suggested that anticipatory and predictive control are proactive strategies based on past experience, which can help participants maintain body stability during locomotion [18]. Therefore, one can predict that participants may change their step length to achieve a postural modification at initial contact using the assumption that the step length is proportional to the horizontal COM-COP distance at initial contact. Thus, our results imply that the shorter horizontal COM-COP distance is a proactive strategy during UDW. The anterior-posterior displacement of the pelvis can be reasonably predicted using the stride frequency and stride length [32]. Therefore, the previous step length and the initial contact position of the trailing limb can be identified if we assume that the previous step length is proportional to the horizontal COM-COP distance at the initial contact of the leading limb and if we know the location of the initial contact position of the leading limb. We further investigated whether the initial contact position of the supporting limb differs between UDW and UW. There were no significant differences between the tasks. Therefore, we expected a shorter step length in the previous step during UDW than that during UW. However, further studies are needed to clearly identify the difference in the proactive strategies that control the COM position relative to the COP at initial contact during UDW and UW.

As our study found that different motor controls were needed to reduce the COM velocity in the propulsion phase during UDW than during UW to decrease the forward momentum of the body, retraining not only in continuous slope walking but also in transition walking on differently sloped terrains might be necessary for walking rehabilitation in patients with neurological or musculoskeletal disorders. In addition, our study found that certain performances at subphases were important to control walking on slopes. To ensure better walking rehabilitation on slopes in the future, the focus should be a thorough assessment of spatiotemporal parameters during subphases of a gait cycle.

A limitation of the current study is that the only participants were healthy young males. Thus, the findings obtained cannot be directly applied to all populations. However, as the fall incidence among women and older adults is generally greater than that of men, young males were chosen as the baseline. Additionally, the angles of both the uphill slope and the downhill slope might influence the findings. Further investigation with a wider variety of slope angles is warranted to understand the relative importance of the slope angle on the transition strategy. In addition, further study of elderly populations or patients with neurological and musculoskeletal disorders using the current experimental protocol is needed to reach more clinically relevant conclusions.

## 5. Conclusions

A slower vertical velocity of the COM during the propulsion phase was observed during UDW; the slower vertical velocity of the COM decreases the body's forward momentum and is able to prevent a forward fall. Decreased positive ankle joint power and work and increased negative knee joint power and work seem to be utilized to decrease the vertical velocity of the COM. Longer-duration *T*_PP_ and *T*_2_ phases are needed to decrease the vertical velocity of the COM. A shorter horizontal COM-COP distance at initial contact helps the participants have shorter *T*_1_ durations and longer *T*_PP_ and *T*_2_ durations during UDW. A modification of the horizontal COM-COP distance at initial contact is required to achieve a slower vertical COM velocity during the propulsion phase during UDW than during UW.

## Figures and Tables

**Table 1:** Mean (standard deviation) of duration, COM and COP movement parameters during UW and UDW.

Parameter	UW	UDW	*p* value	UW	UDW	*p* value
*Stance time (sec)*	0.87 (0.13)	0.91 (0.11)	0.183			
*Duration of subphase (%)*						
Loading response (TLS)	13.1 (3.0)	11.3 (2.8)	0.103			
Midstance (TMS)	61.6 (3.8)	57.1 (10.1)	0.138			
Propulsion (TPP)	25.3 (3.4)	31.5 (10.5)	0.044^∗^			
*T*_1_	62.8 (3.1)	53.3 (4.6)	<0.01^∗^			
*T*_2_	23.3 (6.4)	38.7 (9.5)	<0.01^∗^			
*Distance COM-COP (m)*						
At initial contact	−0.38 (0.04)	−0.34 (0.04)	0.024^∗^			
At foot flat	−0.29 (0.07)	−0.25 (0.04)	0.081			
At heel off	0.08 (0.08)	0.06 (0.10)	0.557			
At toe off	0.22 (0.10)	0.33 (0.05)	<0.01^∗^			
*Displacement (m)*	*COM_horizontal_*			*COM_vertical_*		
Total stance	0.84 (0.06)	0.90 (0.04)	<0.01^∗^	0.16 (0.03)	0.10 (0.03)	<0.01^∗^
*D *_1_	N.A.			0.14 (0.02)	0.13 (0.02)	0.037^∗^
*D *_2_	N.A.			0.04 (0.02)	0.02 (0.01)	<0.01^∗^
*Velocity (m/s)*	*COM_horizontal_*			*COM_vertical_*		
Total stance	1.01 (0.10)	1.02 (0.13)	0.861	0.19 (0.04)	0.11 (0.03)	<0.01^∗^
Loading response	1.31 (0.13)	1.20 (0.12)	0.012^∗^	0.17 (0.09)	0.32 (0.09)	<0.01^∗^
Midstance	0.92 (0.08)	0.91 (0.13)	0.882	0.22 (0.05)	0.16 (0.09)	0.019^∗^
Propulsion	1.10 (0.14)	1.18 (0.19)	0.23	0.17 (0.12)	0.07 (0.05)	<0.01^∗^
*Velocity (m/s)*	*COP_anterior-posterior_*			*COP_medial-lateral_*		
Total stance	0.27 (0.04)	0.22 (0.05)	<0.01^∗^	0.03 (0.03)	0.03 (0.04)	0.66
Loading response	0.55 (0.23)	0.49 (0.27)	0.47	0.20 (0.14)	0.24 (0.25)	0.5
Midstance	0.23 (0.07)	0.22 (0.06)	0.44	0.02 (0.01)	0.03 (0.03)	0.26
Propulsion	0.31 (0.09)	0.20 (0.05)	<0.01^∗^	0.06 (0.04)	0.04 (0.03)	0.25

*Note*. The asterisk (∗) represents a significant difference between continuous uphill walking and up-down transition on a triangle-shaped slope (*p* < 0.05). *N.A.: not applicable*.

**Table 2:** Mean (standard deviation) of ankle and knee joint peak power and work during UW and UDW.

Parameter	UW	UDW	*p* value
*Power (W/(BW * ^∗^ * Ht))*			
Peak positive ankle power	0.20 (0.05)	0.12 (0.04)	<0.01^∗^
Peak negative ankle power	0.03 (0.02)	0.05 (0.03)	0.271
Peak positive knee power	0.14 (0.06)	0.12 (0.04)	0.106
Peak negative knee power	0.17 (0.09)	0.29 (0.10)	<0.01^∗^
*Work (J/(BW * ^∗^ * Ht))*			
Positive ankle work	0.023 (0.013)	0.013 (0.004)	<0.01^∗^
Negative ankle work	0.009 (0.004)	0.006 (0.003)	0.078
Positive knee work	0.030 (0.008)	0.027 (0.006)	0.065
Negative knee work	0.022 (0.011)	0.034 (0.009)	<0.01^∗^

*Note.* The asterisk (∗) represents a significant difference between continuous uphill walking and up-down transition on a triangle-shaped slope (*p* < 0.05).

## Data Availability

The data that support the findings of this study are available from the corresponding author upon reasonable request.
